# Comparison of the pre-treatment functional MRI metrics’ efficacy in predicting Locoregionally advanced nasopharyngeal carcinoma response to induction chemotherapy

**DOI:** 10.1186/s40644-021-00428-0

**Published:** 2021-11-10

**Authors:** Da-wei Zhao, Wen-jun Fan, Ling-ling Meng, Yan-rong Luo, Jian Wei, Kun Liu, Gang Liu, Jin-feng Li, Xiao Zang, Meng Li, Xin-xin Zhang, Lin Ma

**Affiliations:** 1 Medical School of Chinese PLA, No.28 Fuxing Road, Beijing, 100853 China; 2 Department of Radiology, Pingjin Hospital, Characteristic Medical center of Chinese People’s Armed Police Force, Tianjin, China; 3 Department of Radiation Oncology, First Medical Center of Chinese PLA General Hospital, Beijing, China; 4 Affiliated Foshan Maternity & Child Healthcare Hospital, Southern Medical University, Foshan, China; 5 Armed Police Forces Corps Hospital of Henan Province, No.1 Kangfu Road, Zhengzhou, 450052 China; 6 Department of Otolaryngology, First Medical Center of Chinese PLA General Hospital, Beijing, China; 7 Department of Radiology, First Medical Center of Chinese PLA General Hospital, Beijing, China

**Keywords:** Functional magnetic resonance imaging, Magnetic resonance imaging, Induction chemotherapy, Nasopharyngeal carcinoma

## Abstract

**Background:**

Functional MRI (fMRI) parameters analysis has been proven to be a promising tool of predicting therapeutic response to induction chemotherapy (IC) in nasopharyngeal carcinoma (NPC)**.** The study was designed to identify and compare the value of fMRI parameters in predicting early response to IC in patients with NPC.

**Methods:**

This prospective study enrolled fifty-six consecutively NPC patients treated with IC from January 2021 to May 2021. Conventional diffusion weighted imaging (DWI), diffusion kurtosis imaging (DKI), intravoxel incoherent motion (IVIM) and dynamic contrast-enhanced magnetic resonance imaging (DCE-MRI) protocols were performed before and after IC. Parameters maps (ADC, MD, MK, D_slow_, D_fast_, PF, K^trans^, V_e_ and K_ep_) of the primary tumor were calculated by the Functool post-processing software. The participants were classified as responding group (RG) and non-responding group (NRG) according to Response Evaluation Criteria in Solid Tumors 1.1. The fMRI parameters were compared before and after IC and between RG with NRG. Logistic regression analysis and ROC were performed to further identify and compare the efficacy of the parameters.

**Results:**

After IC, the mean values of ADC(*p* < 0.001), MD(*p* < 0.001), D_slow_(*p* = 0.001), PF(*p* = 0.030) and V_e_(*p* = 0.003) significantly increased, while MK(*p* < 0.001), D_fast_(*p* = 0.009) and K_ep_(*p* = 0.003) values decreased dramatically, while no significant difference was detected in K^trans^(*p* = 0.130). Compared with NRG, ADC-pre(*p* < 0.001), MD-pre(*p* < 0.001) and D_slow_-pre(*p* = 0.002) values in RG were lower, while MK-pre(*p* = 0.017) values were higher. The areas under the ROC curves for the ADC-pre, MD-pre, MK-pre, D_slow_-pre and PRE were 0.885, 0.855, 0.809, 0.742 and 0.912, with the optimal cutoff value of 1210 × 10^− 6^ mm^2^/s, 1010 × 10^− 6^ mm^2^/s, 832 × 10^− 6^, 835 × 10^− 6^ mm^2^/s and 0.799 respectively.

**Conclusions:**

The pretreatment conventional DWI (ADC), DKI (MD and MK), and IVIM (D_slow_) values derived from fMRI showed a promising potential in predicting the response of the primary tumor to IC in NPC patients.

**Trial registration:**

This study was approved by ethics board of the Chinese PLA General Hospital, and registered on January 30, 2021, in Chinese Clinical Trial Registry (ChiCTR2100042863).

**Supplementary Information:**

The online version contains supplementary material available at 10.1186/s40644-021-00428-0.

## Background

Nasopharyngeal carcinoma (NPC) is a common malignancies that arises from nasopharyngeal epithelial tissues and has remarkable epidemiological features including regional, racial and familial aggregations, more than 70% of new cases are in east and southeast Asia [1]. Due to the tumor’s deep-seated location, almost 80% patients failed to be diagnosed early until it is at the advanced stage [2].

Although the efficacy and safety of combined chemoradiotherapy in locoregionally advanced NPC made it to be the standard treatment protocol [3], the platinum-based induction chemotherapy (IC) followed by concurrent chemoradiotherapy produced superb outcomes for patients with stage III or IVA/B NPC [4, 5]. Moreover, IC has also been proven to be beneficial for downstaging, thus reduction of tumor size eventually contributes to protection of organ at risks, improving quality of life of NPC [6]. Unfortunately, the patient’s response to IC seems to be variable widely, not all of them can response positively. So accurately predicting response to IC is vital for prognosis and subsequently management [4], specificity for patients with limited and poor response outcomes if it was available prior to the complement of IC.

The advances in imaging modalities have led to improving the ability of diagnosing NPC [7], predicting and assessing tumor responding to treatment [8–12]. The apparent diffusion coefficient (ADC) which represents cellularity and interstitial water mobility is calculated by diffusion weighted imaging (DWI) using mono-exponential model reflecting Gaussian diffusion. ADC has been proven to be a valuable technique to accurately predict therapeutic response to IC in head and neck squamous cell carcinoma [9]. In comparison with DWI, diffusion kurtosis imaging (DKI) potentially provides more information about the underlying microstructure using a polynomial model, reflecting both Gaussian and non-Gaussian diffusion properties [13], representing the interaction between molecules with intercellular compounds and cell membranes [13, 14]. Previous studies have suggested that DKI can be used to evaluate curative effect of IC in malignancies [9–12, 15, 16]. Intravoxel incoherent motion (IVIM) diffusion weighted model, using multiple b values and bi-exponential fitting equation, can quantify and discriminate pure water molecular diffusion and microcirculatory perfusion of the tissue [17, 18]. Several studies have investigated potential value of the parameters derived from IVIM in assessing and predicting its radiotherapy or chemotherapy response [8, 10, 19–21]. Dynamic contrast-enhanced magnetic resonance imaging (DCE-MRI), a functional imaging modality, was identified as a valuable tool of reflecting tumor angiogenesis density, vascular permeability and tumor neo-angiogenesis blood flow [22, 23]. DCE-MRI therefore become prevalent in studies on the prediction of curative effect of malignancies [24–30].

Although there have been studies on investigating the value of single fMRI techniques in assessing and predicting effect of chemotherapy in tumor. However, to our knowledge, most of these studies focused predominantly on the tumors occur in other locations rather than nasopharynx. Moreover, it is worth mentioning that there has been no research to date was designed to compare the value of conventional DWI, DKI, IVIM and DCE-MRI in predicting treatment response of IC in locally advanced NPC. Therefore, the study was aimed to combined DWI, DKI, IVIM and DCE-MRI techniques simultaneously to identify and compare the value of multiple-parameters in predicting early IC response of NPC for the first time.

## Methods

### Patient population and induction chemotherapy regimens

The prospective single-center study protocol was approved by the Institution Review Board of our hospital (Clinical Trails Registration number was ChiCTR2100042863), and all participants signed a written informed consent. From January 2021 to May 2021, fifty-nine consecutively patients pathologically diagnosed NPC were prospectively enrolled.

The inclusion criteria were as follows: (1) histological diagnosis of nasopharyngeal carcinoma (according to AJCC 8th Head and Neck Tumor Staging Criteria: T1-4N2-3M0 but not T1-2N1M0, T3-4N0-1M0, as patients with locoregionally advanced NPC were more likely to receive IC in our center); (2) aged ranges from 18 to 70; (3) KPS ≥70 or ECOC 0–1; (4) be candidate to DCE-MR scan. (5) without any malignancies or anticancer treatment ever; (6) written informed consent. The exclusion criteria were as follows: (1) contraindications to IC; (2) MRI contraindications: metal implants such as dentures and prostheses in the mouth; (3) bad quality of obtained images constrained further analysis. All participants were asked to receive two cycles IC, docetaxel (70 mg/m^2^ on day 1) or Paclitaxel-albumin (260 mg/m^2^ on day 1), cisplatin (P) 40 mg/m^2^ on days 1 and 2. Totally 3 cases were excluded, 2 of them quitted as a result of chemotherapy related toxicities and the third one quitted due to Claustrophobia. There was no excluded case due to bad image quality.

### Functional MRI techniques

All MRI exams were performed on a 3.0 T MR scanner (Signa HDx, GE Healthcare, Milwaukee, WI, USA). Each patient underwent MR imaging within 3 days before the IC and 21–24 days after the second cycle of IC. The MRI protocols consisted of conventional DWI, DKI, IVIM and DCE-MRI sequences, and all of which were implemented in the same examination.

Prior to DWI images, conventional MRI sequences, including axial, sagittal and coronal T2-weighted 2D turbo spin-echo images were obtained with a 16-channal neurovascular head and neck array coil. Subsequently, axial DWI was acquired using a single shot echo-planar imaging sequence, with b value of 0 and 600 mm^2^/s. The axial DKI sequence was performed using a single-shot spin echo echo planar imaging sequence with fast suppression. And the diffusion gradients were applied in three orthogonal gradient diffusion directions. The IVIM was acquired using a prototyped integrated specific slice dynamic Shim (iShim) sequence, the parameters of which were identical to those for DKI, except for using the multiple b values (detailed information was summarized in Additional file 1). DCE-MRI was acquired using FLASH 3D gradient echo sequence [31], to obtain four series of unenhanced images and thirty-one series of enhanced images without any delay after intravenous injection of 0.2 ml/kg of the contrast agent (Gd-TPA, Magnevist, Bayer Schering, Berlin, Germany). The automatic syringe pump was applied to inject the contrast agent, with a flow rate of 2 ml/s, followed by a 20 ml saline flush at the identical rate. The acquisition time of DWI, DKI, IVIM and DCE-MRI were 2:00, 4:09, 2:44 and 4:44 min respectively. After that, the axial, sagittal and coronal post-contrast T1 weighted 2D turbo spin-echo images with fat saturation were acquired in the same position as the T2WI. (Detailed information was summarized in Additional file 1).

### Image analysis

The independent Linux workstation (Advantage Workstation version 4.6, GE Healthcare) was used to process the data. Pixel-wise ADC maps were calculated by a two-variable linear least-square method on the basis of a mono-exponential model, using the following equation [14]: S_i_ = S_0_× exp. (−b_i_ × ADC). The S_i_ means the MRI signal intensity at the diffusion weighting b_i_, while the S_0_ represents that of non-diffusion weighted.

The DKI parameter maps were obtained using the post-processed Functool software. In comparison with the mono-exponential equation, the DKI model yielded two variables while S_0_ is known, according to the following equation [13, 14]: S_i_ = S_0_ × exp.(−b_i_ × D+ $\frac{1}{6}$ b_i_^2^ × D^2^ × K). with S_0_, D, and K as fitting variables, where S_i_ is the signal at a particular b value, S_0_ is the baseline signal without diffusion gradient [16]. Accordingly, D is diffusivity, K describes peakedness of a probability of water distribution [13, 14]. The parameter MD is the diffusion coefficient in normal diffusion after correcting the non-Gaussian effect, while MK reflects non-Gaussian diffusion behavior. MK as the non-Gaussian component may depict the inhomogeneity of diffusion that cannot be measured with conventional DWI.

After the IVIM raw data was transferred to the Linux workstation, and the parameters including D_slow_, D_fast_ and PF were calculated by MADC prototype software in the Functool software package. The assumption of the IVIM model was based on the translation movements at voxel levels. IVIM signal attenuation is the sum of the tissue and blood component, taking the shape of biexponential decay [18]: S_i_/S_0_ = (1-*f*) × exp.(−b × D_slow_) + exp.(−b_i_ × D_slow_ + D_fast_). S_i_ means the signal intensity (SI) with diffusion gradient b_i_, S_0_ represented SI with the diffusion gradient was 0. D_slow_ means the true diffusion representing pure molecular diffusion (mm^2^/s), while D_fast_ represents pseudo diffusion coefficient as reflected by perfusion relative diffusion or incoherent microcirculation (mm^2^/s), PF acted as fractional perfusion related to microcirculation [18].

The DCE parameter maps including K^trans^, V_e_, K_ep_, were calculated using two-compartment model regards the tissue and plasma as two compartments [23]. The transport was determined by volume transfer constant: K^trans^ (from the blood plasma to extracellular-extravascular space) and K_ep_ (from extracellular-extravascular space to the blood plasma). The parameter v_e_ was defied as EES fractional volume and was calculated by the following equation: V_e_ = K^trans^/K_ep_.

**Fig. 1:**
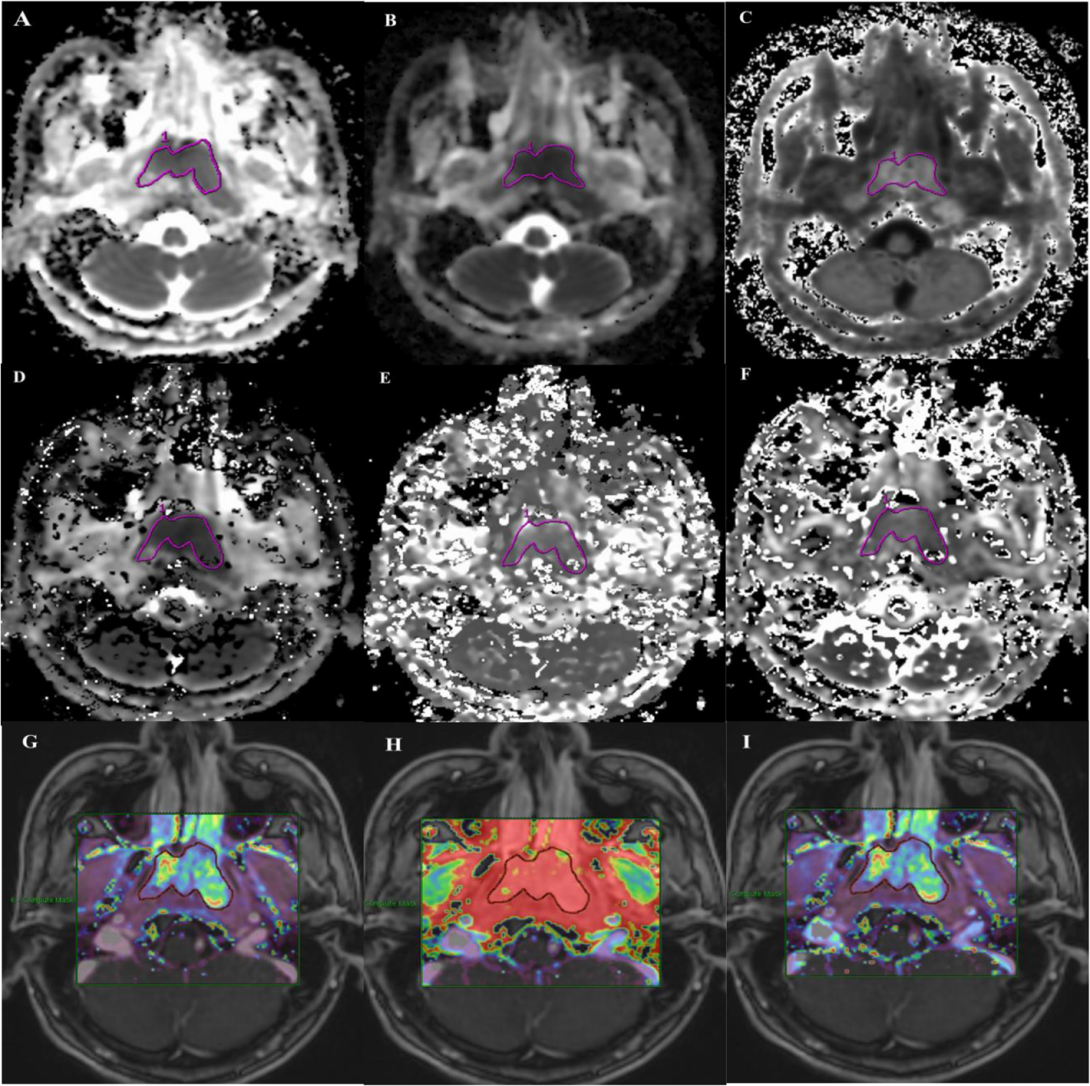
Pre-IC, (A) ADC, (B) MD, (C) MK, (D) Dslow, (E) Dfast, (F) PF, (G) Ktrans, (H) Ve, (I) Kep maps derived from fMRI were obtained in a 19-year-old man with nasopharyngeal carcinoma

The largest slice of the target lesions on the axial plane was identified by two radiologists (15 years of experience in head and neck imaging). Region of interest (ROI) of the target lesions were manually drawn on conventional DWI/DKI/IVIM/DCE-MRI parameter maps with the principle of encompassing the whole tumor area (as shown in Fig. 1). The time required for analysis of every single imaging varied from 3 to 5 min. Overall, ADC values took the shortest time, while longer time was required for the post-processing of DCE-MRI. The changes of the parameters before and after IC were calculated as followings equation: △(parameters) = post (parameters) –pre (parameters); △(parameters)% = △(parameters)/pre (parameters)%. Tumor volume was calculated as follows: Volume = area of lesion× (thickness of slice + interstice gap).

### Response evaluation

The target lesions were classified as responding group (complete or partial response) and non-responding group (stable or progressive disease) after two cycles of chemotherapy, assessed by Response Evaluation Criteria in Solid Tumors (RECIST Version1.1). The primary tumor was identified as the only target lesion to be assessed according to RECIST 1.1, the pathologically enlarged cervical lymph nodes were not evaluated. The regression ratio and the tumor volume reduction ratio was calculated as the following equation: △(Diameters/Volume) = post (Diameters /Volume) – pre (Diameters/Volume); △(Diameters/Volume)% = △(Diameters/Volume) /pre (Diameters/Volume)%.

### Statistical analysis

Categorical data was compared by chi-square test, correction for continuity or Fisher’s exact test. Before and after treatment, mean value of these parameters were compared using Paired t test or Wilcoxon rank-sum test (according to normality of data distributed). Independent-samples t test or Mann-Whitney U test (according to normality of data distributed) was performed to compare the mean value of the parameters and changes of parameters after treatment between RG and NRG. Logistic regression analysis was applied to fit the significant parameters to generate a new predictive factor (PRE). ROC curve analyses were performed to further identify and compare the efficacy of the significant parameters in terms of the value of predicting IC outcomes. All data were analyzed by SPSS 26.0 and Medcalc. A *p* value less than 0.05 was considered statistically significant.

## Results

### Participants and tumor characteristics

A totally 56 participants were enrolled in the study, including 46 males and 10 females, mean age was 45.54 ± 12.84 years (range from 18 to 67). Subsequent to two cycles of IC, the regression ratio in diameters of the primary tumor was 10.30 ± 10.12%, and tumor volume’s reduction ratio was 52.61 ± 23.63%. 36 patients were assigned to RG, of the remaining 20 were categorized as NRG. The reduction rate of tumor diameters in RG (49.24%) was significantly higher than NRG (18.62%)(*p < 0.001*), and the same trend was observed for volume changes (64.98 ± 17.77% and 30.35 ± 14.94%, respectively) (*p < 0.001*). No significant difference was found in age, sex, T, N, pathological classification and chemotherapy regimens between RG and NRG (Table 1).

**Table 1:** Patient demographic data and clinical characteristics of responding group and nonresponding group

	RG(***n*** = 36)	NRG(***n*** = 20)	t/χ^**2**^	***p***
**Age**	43.75 ± 13.14	48.75 ± 11.92	1.41	0.165
**Gender**			0.003	0.959
Male	29 (80.6%)	17 (85.0%)		
Female	7 (19.4%)	3 (15.0%)		
**AJCC T stage**			2.716	0.444
T1	2 (5.6%)	0 (0%)		
T2	18 (50.0%)	7 (35.0%)		
T3	10 (27.8%)	7 (35.0%)		
T4	6 (16.7%)	6 (30.0%)		
**A CC N stage**			2.869	0.406
N0	1 (2.8%)	1 (5.0%)		
N1	5 (13.9%)	6 (30.0%)		
N2	16 (44.4%)	6 (30.0%)		
N3	14 (38.9%)	7 (35.0%)		
**Pathological classification**			3.283	0.224
non-cornification undifferentiated	19 (52.8%)	7 (35.0%)		
low differentiated	15 (41.7%)	9 (45.0%)		
moderately differentiated	2 (5.6%)	4 (20.0%)		
**IC regimes**			1.344	0.246
Docetaxel + Cisplatin	27 (75%)	12 (60%)		
Paclitaxel-albumin + Cisplatin	9 (25%)	8 (40%)		

Note: data represents the number of patients and data in parentheses are percentages. Abbreviations: IC = induction chemotherapy. Abbreviations: RG: responding group; NRG: nonresponding group; IC: induction chemotherapy

### Comparison of fMRI parameters before and after IC

**Fig. 2:**
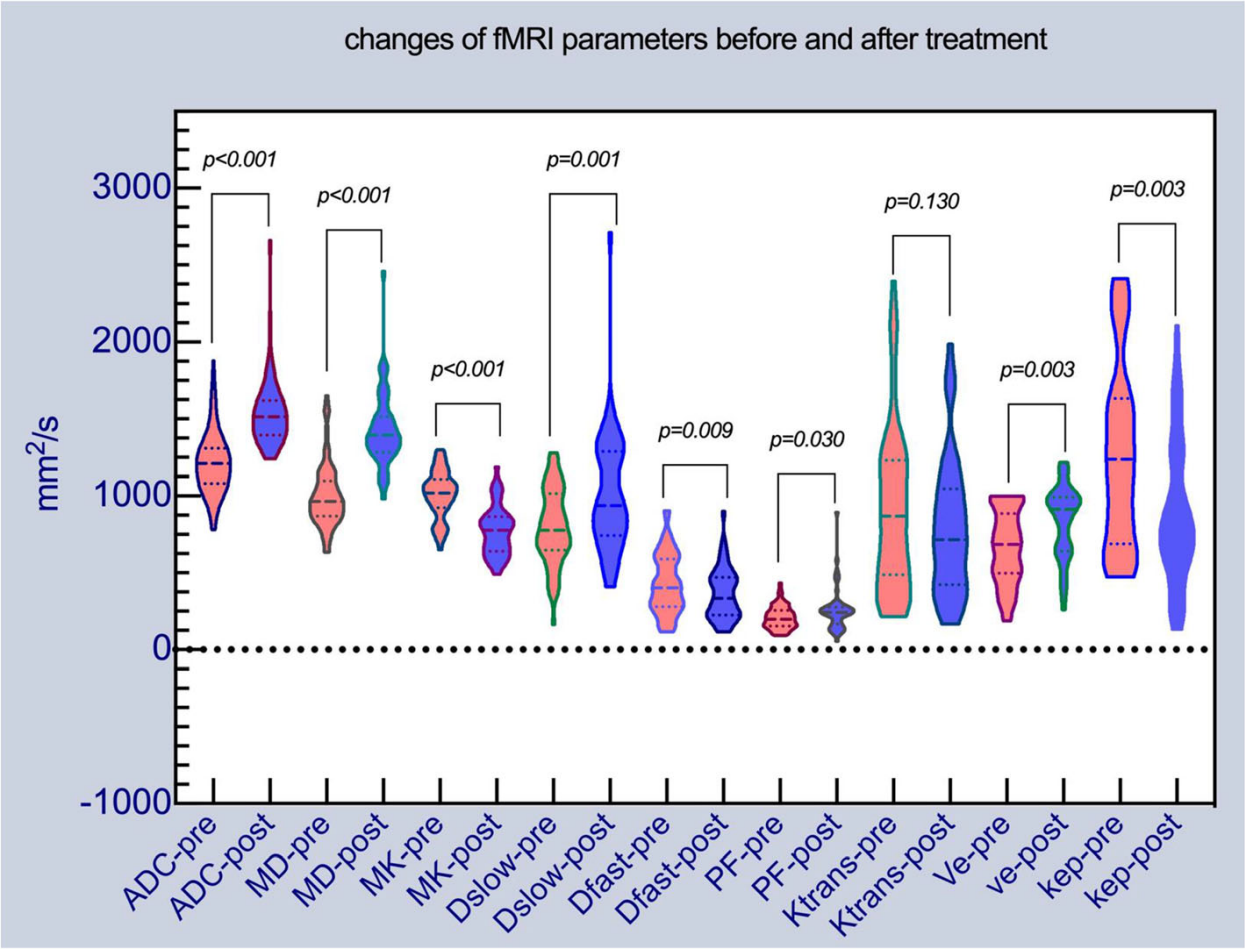
Violin distribution of the fMRI parameters before and after IC. Red violin figures represent pre-treatment fMRI values, and blue violin figures represent post-treatment fMRI values

There were statistically significant differences in ADC, MD, MK, D_slow_, D_fast_, PF, V_e_ and k_ep_ except for K^trans^ before and after IC. After treatment, the mean values of ADC, MD, D_slow_, PF and V_e_ significantly increased, while MK, D_fast_ and K_ep_ values decrease dramatically (Table 2, Fig. 2).

**Table 2:** The comparison of fMRI parameters before and after IC

	Pre-IC	Post-IC	***t/Z***	***p***
**ADC (× 10**^**−6**^ **mm**^**2**^**/s)**	1238.43 ± 211.932	1549.84 ± 242.527	−11.441	.000^♦^
**MD (×10**^**−6**^ **mm**^**2**^**/s)**	1012.50 ± 223.836	1432.61 ± 252.577	−12.603	.000^♦^
**MK (×10**^**−6**^**)**	1020.00 (183)	779.00 (226)	−5.485	.000^Δ^
**D**_**slow**_ **(×10**^**−6**^ **mm**^**2**^**/s)**	778.00 (370)	937.00 (548)	−3.227	.001^Δ^
**D**_**fast**_ **(× 10**^**−4**^ **mm**^**2**^**/s)**	410.59 ± 181.513	363.16 ± 170.045	2.729	.009^♦^
**PF (×10**^**−4**^**)**	198.50 (103)	243.00 (109)	−2.165	.030^Δ^
**K**^**trans**^**(×10**^**−3**^**/min)**	920.43 ± 549.753	790.48 ± 445.798	1.543	.130^♦^
**V**_**e**_ **(×10**^**−3**^**)**	686.18 ± 242.582	838.14 ± 235.443	−3.205	.003^♦^
**K**_**ep**_ **(×10**^**−3**^**/min)**	1239.50 (945)	805.00 (679)	−3.019	.003^Δ^

Abbreviations: IC: induction chemotherapy; ADC: apparent diffusion coefficient(×10^−6^ mm^2^/s), MD: mean diffusion (× 10^− 6^ mm^2^/s), MK: mean kurtosis (× 10^− 6^), D_slow_: true diffusion coefficient(× 10^− 6^ mm^2^/s), D_fast_: pseudo diffusion coefficient (× 10^− 4^ mm^2^/s), PF: perfusion fraction (× 10^− 4^), K^trans^: volume transfer constant (× 10^− 3^/min), Ve: extracellular extravascular space (× 10^− 3^), K_ep_: rate constant(× 10^− 3^/min). Statistically significant differences determined by Paired t test are marked with “^♦^” (data were reported as mean values ± standard error), while Wilcoxon rank-sum test are marked with” ^Δ^” (data were presented as median (interquartile range))

### Comparison of fMRI parameters between RG and NRG

**Fig. 3:**
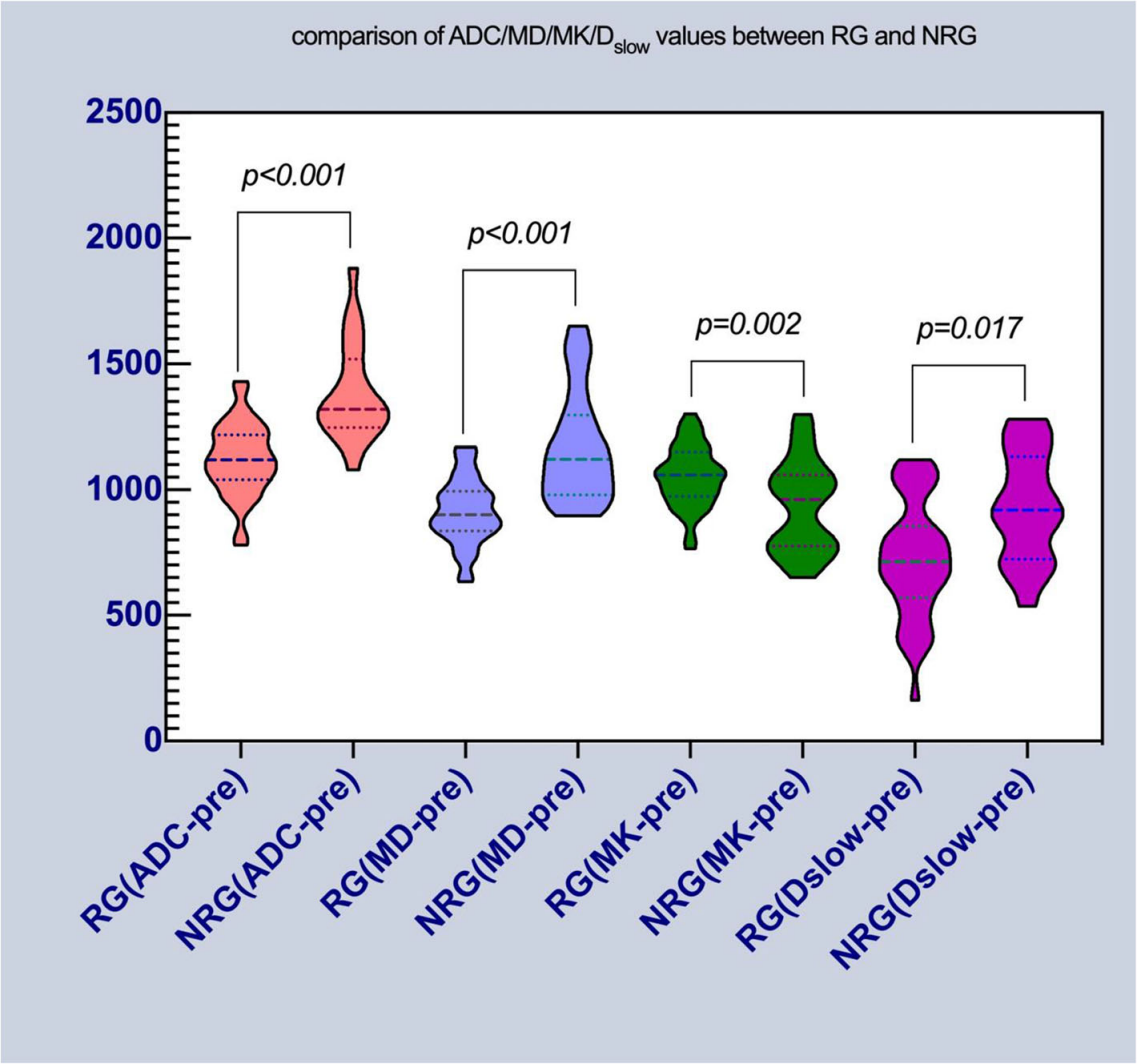
Violin distribution of ADC/MD/MK/D_slow_ between RG and NRG. Note: pink represents parameter of ADC; light blue represents MD; green represents MK; red represents D_slow_

Statistically significant differences were identified in ADC-pre, ADC-post, ΔADC%, MD-pre, ΔMD, ΔMD%, MK-pre, ΔMK, ΔMK%, D_slow_-pre between RG and NRG. The results, as shown in Table 3, Fig. 3 (see Additional file 2), also indicate that the mean value of ADC-pre(*p < 0.001*), MD-pre(*p < 0.001*) and Dslow-pre(*p = 0.002*) in RG were lower than those in NRG, in contrast to the value of MK-pre(*p = 0.017*).

**Table 3:** The fMRI parameters with statistical differences between RG and NRG

	RG(***n*** = 36)	NRG(***n*** = 20)	***t/U***	***p***
**D-post (cm)**	1.18 (0.68)	1.94 (0.98)	113.500	0.000^Δ^
**ΔD (cm)**	−1.27 (0.59)	−0.4 (0.54)	52.000	0.000^Δ^
**ΔD%**	−49.24%(24.14)	−18.62 (11.71)	0.000	0.000^Δ^
**V –post (cm**^**3**^**)**	4.80 (8.97)	11.68 (10.08)	177.000	0.002^Δ^
**ΔV (cm**^**3**^**)**	−10.48 (9.11)	−4.92 (4.17)	152.000	0.000^Δ^
**ΔV %**	−64.98 ± 17.77	−30.35 ± 14.94	7.377	0.000^♦^
**ADC-pre (×10**^**−6**^ **mm**^**2**^**/s)**	1119.58 ± 144.06	1386.25 ± 198.23	5.790	0.000^♦^
**ADC-post (×10**^**−6**^ **mm**^**2**^**/s)**	1410.00 (258)	1557.00 (320)	106.000	0.002^Δ^
**ΔADC%**	33.81 (30)	18.14 (30)	206.500	0.430^Δ^
**MD-pre (×10**^**−6**^ **mm**^**2**^**/s)**	902.00 (150)	1121.00 (331)	104.500	0.000^Δ^
**ΔMD (×10**^**− 6**^ **mm**^**2**^**/s)**	504.46 ± 106.51	318.90 ± 242.72	−3.023	0.004^♦^
**ΔMD%**	58.56 ± 20.49	28.61 ± 21.77	−4.666	0.000^♦^
**MK-pre (×10**^**−6**^**)**	1052.25 ± 122.02	935.10 ± 185.34	−2.538	0.017^♦^
**ΔMK (×10**^**−6**^**)**	492.00 (217)	305.00 (144)	129.000	0.009^Δ^
**ΔMK%**	60.92 (20)	28.17 (14)	131.000	0.010^Δ^
**D**_**slow**_**-pre (×10**^**−6**^ **mm**^**2**^**/s)**	724.50 ± 235.92	933.85 ± 219.95	3.258	0.002^♦^

Abbreviations: D: diameters of tumor (cm); V: volume of tumor (cm^3^); ADC: apparent diffusion coefficient (×10^−6^ mm^2^/s), MD: mean diffusion (× 10^− 6^ mm^2^/s), MK: mean kurtosis (× 10^− 6^), D_slow_: true diffusion coefficient (× 10^− 6^ mm^2^/s). Statistical analysis was performed using the independent-samples t test were marked with “^Δ^” (data were reported as mean values ± standard error), while using Mann-Whitney U test were marked with” ^♦^”(data were presented as median (interquartile range))

### The diagnostic performance of MRI parameters

**Fig. 4:**
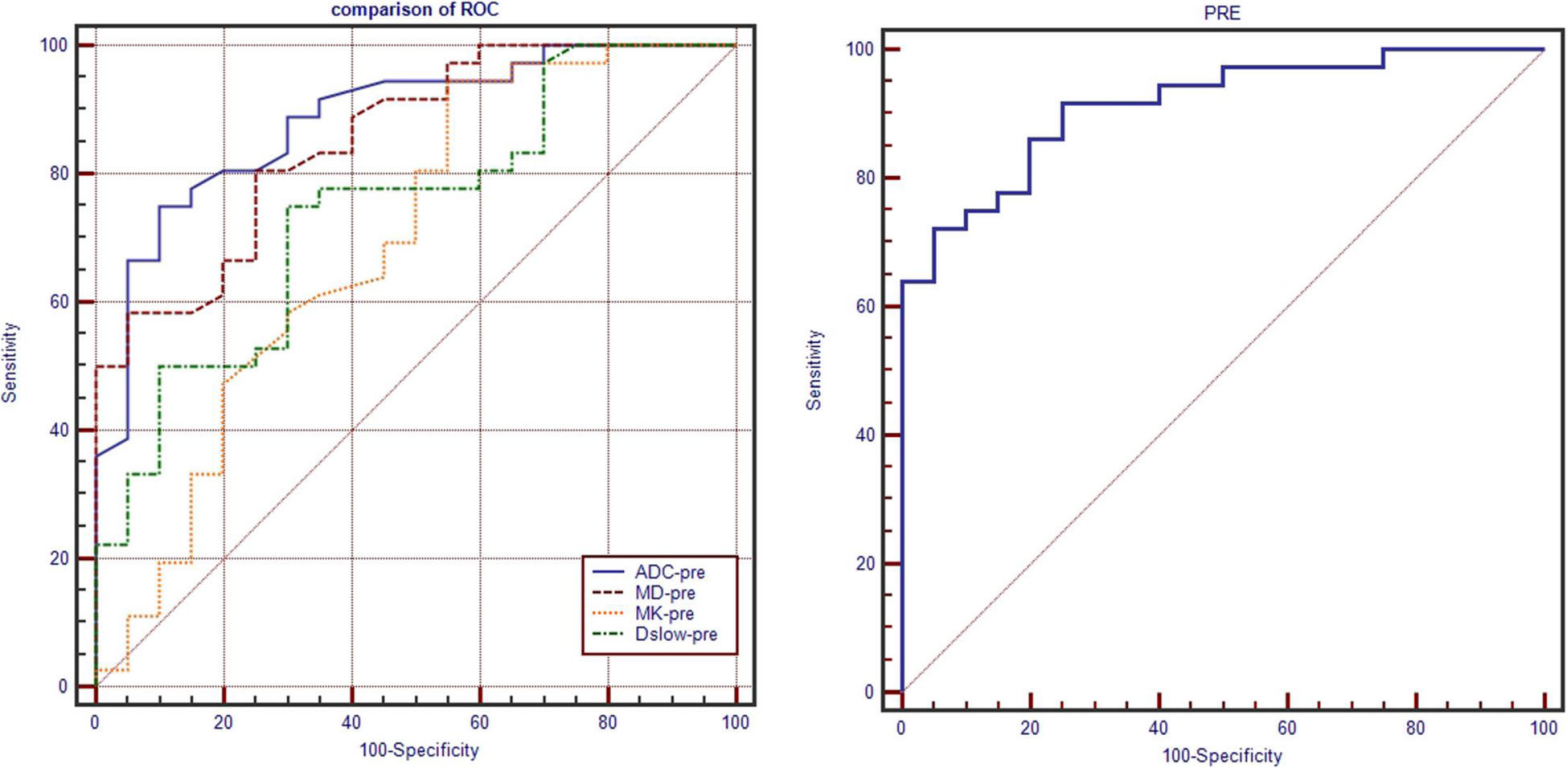
ROC of fMRI parameters in predicting the treatment response of IC

The areas under the ROC curves for parameters ADC-pre, MD-pre, MK-pre and D_slow_-pre were 0.885, 0.855, 0.809 and 0.742 respectively, with the optimal cutoff value of ADC-pre (1210 × 10^− 6^ mm^2^/s), MD-pre (1010 × 10^− 6^ mm^2^/s), MK-pre (832 × 10^− 6^), D_slow_-pre (835 × 10^− 6^ mm^2^/s) (Table 4, Fig. 4). Statistically significant difference was only found between ADC-pre with MK-pre (*p* = 0.012), MD-pre with MK-pre (*p* = 0.016) (see Additional file 3). The PRE was generated by fitting the above four metrics, with AUC of 0.912, the sensitivity of 72.22% and specificity of 95.00% (Table 4, Fig. 4).

**Table 4:** Diagnostic efficacy of ADC-pre, MD-pre, MK-pre, D_slow_-pre and PRE

	AUC	95%CI	Youden	Cutoff	sensitivity	specificity	+LR	-LR	+PV	-PV
**ADC-pre** (×10^−6^ mm^2^/s)	0.885	(0.772,0.955)	0.650	1210	75.00%	90.00%	7.50	0.28	93.1	66.7
**MD-pre** (×10^−6^ mm^2^/s)	0.855	(0.735,0.935)	0.556	1010	80.6%	75.0%	3.22	0.26	85.3	68.2
**MK-pre** (×10^−6^)	0.692	(0.555,0.809)	0.394	832	94.4%	45.0%	1.72	0.12	75.6	81.8
**D**_**slow**_**-pre** (×10^−6^ mm^2^/s)	0.742	(0.608,0.850)	0.450	835	75.00%	70.00%	2.50	0.36	81.8	60.9
**PRE**	0.912	(0.806,0.971)	0.672	0.799	72.22%	95.00	14.44	0.29	96.3	65.5

Abbreviation: AUC: area under the curve; 95% CI: 95% confidence interval; +LR: positive likelihood ratio; −LR: negative likelihood ratio; +PV: positive predictive value; −PV: negative predictive value

## Discussion

Accurately predicting response to IC might improve outcomes for locally advanced NPC by allocating patients who was insensitive to chemotherapy to non-IC treatment, to avoiding unwanted chemotherapy related toxicities. The present study was set out to analysis and compare the value of fMRI parameters in predicting therapeutic response to IC in locoregionally advanced NPC. And the study found that the parameters derived from fMRI could reflect the changes of tumor microstructure after IC. After treatment, the mean value of ADC, MD, D_slow_, D_fast_, PF and V_e_ significantly increased, while MK and K_ep_ decreased. Perhaps the most important findings were that the mean value of ADC-pre, MD-pre and D_slow_-pre were lower in RG than those in NRG, conversely higher the value of MK-pre was. Furthermore, ROC analysis showed the promising potential of fMRI parameters in discriminating RG from NRG by the indicators of ADC-pre, MD-pre, MK-pre, D_slow_-pre and PRE.

As a functional imaging technique reflecting water diffusion in tissues, DWI has shown a promising value of revealing changes of tumor at the cellular level. ADC derived from DWI and held strong potential to be an imaging biomarker to reflect therapeutic response early [15]. Although it is an “apparent” metrics and has no direct biophysical basis, ADC is thought to be closely related to extracellular space which can be influenced by tissue architectural properties [13]. So, differences of biological character between RG and NRG can be correctly reflected by changes of ADC theoretically. As mentioned in the literature review, high pre-treatment ADC could be predictor of outcomes in patients with HNSCC [9]. Specificity, Prior studies have noted that pretreatment tumor ADC values were supposed to be a noninvasive important prognostic parameter for NPC [32]. According to these findings, low ADC value before treatment was identified as a valuable indicator of good response to IC. It is not hard to follow actually, higher ADC values often means less cellularity, more necrosis in micro-structure and poor perfusion, which can contributed to the lack of chemotherapeutic drugs delivered to the tissue [10].

Our results showed that the mean value of ADC significantly increased after IC. The significant increase of ADC values might result from the chemotherapy associated microstructure necrosis in the tumor, which were in general agreement with literature [11, 20, 33–35]. The current study also found that ADC-pre and ADC-post values is lower in RG than those in NRG, which is consistent with that of prior studies which have demonstrated that good responder group had lower pretreatment ADC values than the poor responder group did [33, 35]. These discoveries corroborated with the ideas of Yu, who suggested that non-residual group also had lower ADC-pre than the residual group [20]. The role of ADC values in predicting treatment response to chemoradiotherapy was also proven in metastatic lymph nodes in patients with NPC [8]. King et al. found that the primary tumor showed significantly lower increase in percentage change of mean ADC for local failure than for local control [34]. These findings support our results in some extent, although some of them used local control or residual as the indicator other than responder. The higher ADC values reflects more micro-necrosis in the tumor, which might led to a poor responses by the lack of perfusion of chemotherapeutic drugs.

DKI is based on a non-Gaussian distribution assumption, which provides an opportunity to get further insights into the actual diffusion of water molecules in vivo [13]. In addition to MD, the introduction of diffusion kurtosis coefficient (MK) facilitates the evaluation of non-Gaussian diffusion behavior and quantitative analysis of the extent of deviation. Our study showed that an increase in MD-pre value, decrease in MK-pre value after IC, and MD-pre values was lower in RG than those in NRG. These could be explained by accelerated expansion of extracellular space and more isotropic lead to rapid decline of MK values [36]. Previous investigations supported our findings. Zheng et al. [12]. proposed that the responding group presented higher baseline MD compared with nonresponding group. And the result was also consistent with that of Chen’s conclusion [11]. Theoretically, the lower MD-pre values indicated a decrease in tumor cell density or an expansion of the extracellular space and more free diffusion of extracellular water which might be beneficial for blood supply as well as chemotherapy drugs transportation. However, both noted that there was no significant difference in MK-pre values between groups, which differed from our findings. The current study observed the fact that pretreatment MK values was higher in RG than that of in NRG might indicated increased irregularity and heterogeneity of tissue microstructure as well as the amount of interface of cellular tissues in RG [13]. In spite of this result has not previously been described in patients of NPC, However, some published articles indicated that pretreatment K values in RG indeed different from that in NRG for other cancers [10, 37].

IVIM reflects the random microscopic motion that occurred in voxel on images of water molecules intra-cellular or extracellular and microcirculation of blood [38]. Compared with conventional DWI, IVIM-MRI can simultaneously obtain information of tumor tissue diffusion and perfusion, and may serve as predictors of effective response [19]. Our results found that D_slow_ and PF values increase, whereas D_fast_ decrease significantly after IC, which can be explained by changes of diffusion and perfusion characteristics in tumor tissue, including dramatically decrease in the cellularity and micro vessels. Similar study have been reported similar conclusions, except for PF-pre values, the reason might be that the duration of 1 cycle IC in that study is too short for the parameter to be detected [39]. D_slow_ reflects the true water diffusion and was related to extracellular spaces. The present study also indicated that D_slow_-pre tend to be a predictor of an effective response, and was consistent with the previous findings [10, 17, 20, 40], which also demonstrated that no perfusion-related parameters could be used to distinguish responders from non-responders.

DCE-MRI allows for probing perfusion and micro-vessel permeability using Tofts Pharmacokinetic analysis of images. Our results also showed that V_e_ values increase, whereas K_ep_ values decrease significantly after IC. K^trans^-pre tended to be higher than K^trans^-post, although there was also no significantly difference before and after IC. This results agreed with previous conclusions that K^trans^, K_ep_ and their ratio V_e_ can be used to quantitative analysis physiological properties of tumor [41]. Besides, these parameters were considered as predictors of therapeutic response in head and neck cancers [42]. Studies have reported that K^trans^ and early changes of K^trans^ were potential markers to predicting response right after one IC cycle for NPC patients [28], and pretreatment primary lesion quantitative DCE-MRI may be valuable in predicting the prognosis for NPC [43]. DCE-MRI were also promising in predicting response to IC in other cancers (esophageal cancer, Breast cancer and cervical squamous cell carcinoma) [25, 27, 44]. In contrast to previous findings, However, no significant differences in K^trans^, K_ep_ and V_e_ were detected among responders and non-responders in the study. These negative findings might be a result of heterogeneity of tumor and the complexity of adjacent structures invaded by the primary tumor. Besides, the poor reproducibility of the parameters produced by DCE-MRI could be another influencing factor. It was reported that the repeatability of the parameters largely depend on body part and imaging sequence and the choose artery for arterial input function measurement [41].

To our knowledge, we present a first attempt to identify and compare the value of fMRI metrics in predicting therapeutic response to IC in NPC patients. We found that the best metrics to discriminate responders from non-responders were ADC-pre and MD-pre. The attempts to combine multiple statistically significant MRI parameters failed to generate stronger predictor over single one. Multivariable Logistic regression analysis showed only ADC-pre was identified as an independent predictor for responders. However, the predictor PRE which was fitted by ADC, MD, MK and D_slow_ using the way of input of Logistic regression analysis, showed a promising value of predicting response to IC, with an area under ROC of 0.912, a sensitivity of 75%, a specificity of 95%, cutoff values of 0.799. These results were in line with those of Wong et al. [33]. Who also claimed that ADC-pre was a powerful predictor of response to IC (AUC: 0.829). As shown in literatures [20, 21, 45], the pretreatment ADC cutoff values ranged from 0.86 to 1.2 to distinguish patients with effective (or locoregional failure) from ineffective (locoregional control) groups. The results reported by Chen et al. [11]. suggested that the MD-pre provided worse diagnostic performance for evaluating response to IC (AUC = 0.593). As reported in prior studies, the AUC of MD-pre for predicting response to IC in NPC was 0.765(reported by Yu [20]) and 0.714 (by Xiao [39]) respectively. Zheng et al. [12]. reported the AUC for DKI was 0.898 (ranged from 0.803 to 0.993). Xiao et al. [39] found the threshold of baseline MD values that best predicted the responders for primary nasopharynx tumors were 0.911 × 10^− 3^ mm^2^/s, which was similar to ours (1010 × 10^− 6^ mm^2^/s). ADC-pre and MD-pre as the reliable indicators of good responders to IC in NPC patients could allow the modification of the chemotherapy regime, indicate the need for a switch to alternative strategies, improving their chance of therapeutic success, and sparing the patients from ineffective treatment burdened by unnecessary toxicities. Given its feasibility in the clinical setting and superiority of efficacy, ADC-pre was more essential for guiding clinical practice and prognosis, especially for patients with limited or poor response outcomes if it was available prior to the complement of IC.

Several limitations of this study need to be acknowledged. First of all, the single-center study with which has its inherent shortcomings. The lack of generalizability of the data could be addressed by explored a multi-center, larger sample study. Secondly, the patients were classified into RG and NRG by RECIST 1.1, however, the method may incorrectly classified patients who response positively to IC into NRG, which attributed to the fact that microstructural changes precede morphological alterations. Attempt to obtain pathological evidence failed to confirm the response to IC with the golden criteria due to unresectability or inoperability of NPC. Finally, the mean value of fMRI parameters can only explain partial property of the target tissues other than reflecting the whole characteristics of tumor. Therefore, further in-depth research is required, and Radiomics of the fMRI parameters-maps for detecting microstructural changes and predicting treatment response to IC is a promising topic in this direction.

## Conclusions

The purpose of the current study was to identify and compare the value of conventional DWI, DKI, IVIM and DCE-MRI in predicting early response to IC in locoregionally advanced NPC. The pretreatment ADC, MD, MK, D_slow_ values emerged as reliable predictive factors of treatment response, which demonstrated that fMRI parameters have a potential to early detect the efficacy of IC in NPC patients, despite the failure of DCE-MRI in predicting curative efficacy. Multicenter radiomics trails are urgently needed to verify our findings.

## Supplementary Information

**Additional file 1.** . MRI standard protocol

**Additional file 2.** . The comparison of fMRI parameters between RG and NRG

**Additional file 3.** . Pair wise comparison of ROC curves

## Data Availability

All data generated or analyzed during this study are included in this published article and its supplementary information files.

## Abbreviations

fMRI: Functional magnetic resonance imaging
NPC: nasopharyngeal carcinoma
IC: induction chemotherapy
RG: responding group
NRG: non-responding group
DKI: diffusion kurtosis imaging
IVIM: intravoxel incoherent motion
DCE-MRI: dynamic contrast-enhanced magnetic resonance imaging
ADC: apparent diffusion coefficient
MD: mean diffusivity
MK: mean kurtosis
D_slow_: pure molecular diffusion coefficient
D_fast_: pseudo diffusion coefficient
PF: fractional perfusion
EES: extravascular extracellular space
K^trans^: volume transfer constant
K_ep_: reflux rate constant between EES and plasma
V_e_: volume fraction of EES
