# Supplementary material for: Comparison of the pre-treatment functional MRI metrics’ efficacy in predicting Locoregionally advanced nasopharyngeal carcinoma response to induction chemotherapy
Source: Cancer Imaging. 2021 Nov 10;21:59. doi: 10.1186/s40644-021-00428-0 (PMC8579637; doi:10.1186/s40644-021-00428-0)
Supplement: Supplementary file 1 — Additional file 1. . MRI standard protocol [file 40644_2021_428_MOESM1_ESM.pdf]

MRI standard protocol.

	T2-weighted	DWI	DKI	IVIM	DCE
**TR**	5299.0	2000.0	3500.0	4000	5.0
**TE**	68.0	67.9	86.8	73.3	1.2
**Slice thickness**	6.0	6.0	6.0	6.0	5.0
**Slice gap**	1.0	1.0	1.0	1.0	1.0
**Fov**(mm^2**)**	180×240	180×240	220×220	220×220	300×300
**Bandwidth**	62.50	250.0	250.0	250.0	125.0
**b values**		(0, 600)	(0,500, 1000,	(0,25,50,75,100,150	
**(**mm^2/s**)**			2000)	,200,500,800, 1000)	
**Time (min)**	4:30	2:00	4:09	2:44	4:44

Abbreviations: DKI: diffusion kurtosis imaging; IVIM: intravoxel incoherent motion; DCE-MRI: dynamic contrast enhanced magnetic resonance imaging;
