# Supplementary material for: Comparison of the pre-treatment functional MRI metrics’ efficacy in predicting Locoregionally advanced nasopharyngeal carcinoma response to induction chemotherapy
Source: Cancer Imaging. 2021 Nov 10;21:59. doi: 10.1186/s40644-021-00428-0 (PMC8579637; doi:10.1186/s40644-021-00428-0)
Supplement: Supplementary file 2 — Additional file 2. . The comparison of fMRI parameters between RG and NRG [file 40644_2021_428_MOESM2_ESM.pdf]

The comparison of fMRI parameters between RG and NRG.

	RG(n=36)	NRG(n=20)	*t/U*	*p*
**D-pre(cm)**	2.58 (1.12)	2.70 (1.14)	351.500	0.627$^{\Delta}$
**D-post(cm)**	1.18 (0.68)	1.94 (0.98)	113.500	0.000$^{\Delta}$
**ΔD(cm)**	-1.27 (0.59)	-0.4 (0.54)	52.000	0.000$^{\Delta}$
**ΔD%**	-49.24%(24.14)	-18.62 (11.71)	0.000	0.000$^{\Delta}$
**V-pre (cm^3)**	16.25 (16.98)	15.73 (8.98)	356.000	0.945$^{\Delta}$
**V –post(cm^3)**	4.80 (8.97)	11.68 (10.08)	177.000	0.002$^{\Delta}$
**ΔV(cm^3)**	-10.48 (9.11)	-4.92 (4.17)	152.000	0.000$^{\Delta}$
**ΔV %**	-64.98±17.77	-30.35±14.94	7.377	0.000$^{\blacklozenge}$
**ADC-pre(×10^{-6} mm^2/s)**	1119.58±144.06	1386.25±198.23	5.790	0.000$^{\blacklozenge}$
**ADC-post(×10^{-6} mm^2/s)**	1410.00 (258)	1557.00 (320)	106.000	0.002$^{\Delta}$
**ΔADC(×10^{-6} mm^2/s)**	336.79±180.34	289.45±178.81	-0.870	0.389$^{\blacklozenge}$
**ΔADC%**	33.81 (30)	18.14 (30)	206.500	0.430$^{\Delta}$
**MD-pre(×10^{-6} mm^2/s)**	902.00 (150)	1121.00 (331)	104.500	0.000$^{\Delta}$
**MD-post(×10^{-6} mm^2/s)**	1385.00 (212)	1406.00 (390)	199.500	0.340$^{\Delta}$
**ΔMD(×10^{-6} mm^2/s)**	504.46±106.51	318.90±242.72	-3.023	0.004$^{\blacklozenge}$
**ΔMD%**	58.56±20.49	28.61±21.77	-4.666	0.000$^{\blacklozenge}$
**MK-pre(×10^{-6})**	1052.25±122.02	935.10±185.34	-2.538	0.017$^{\blacklozenge}$
**MK-post(×10^{-6})**	783.58±180.70	761.70±137.23	-0.445	0.659$^{\blacklozenge}$
**ΔMK(×10^{-6})**	492.00 (217)	305.00 (144)	129.000	0.009$^{\Delta}$

**ΔMK%**	60.92 (20)	28.17 (14)	131.000	0.010$^{\Delta}$
**D_{slow}-pre(×10^{-6} mm^2/s)**	724.50±235.92	933.85±219.95	3.258	0.002$^{\blacklozenge}$
**D_{slow}-post(×10^{-6} mm^2/s)**	875.00 (484)	996.00 (606)	177.500	0.141$^{\Delta}$
**ΔD_{slow}(×10^{-6} mm^2/s)**	252.50 (509)	167.00 (299)	200.000	0.346$^{\Delta}$
**ΔD_{slow}%**	42.23 (70)	15.19 (36)	178.000	0.144$^{\Delta}$
**D_{fast}-pre(×10^{-4} mm^2/s)**	375.00 (174)	433.00 (292)	319.000	0.483$^{\Delta}$
**D_{fast}-post(×10^{-4} mm^2/s)**	385.38±191.83	336.50±139.72	0.286	0.348$^{\blacklozenge}$
**ΔD_{fast}(×10^{-4} mm^2/s)**	-161.64±282.28	-106.85±90.49	-0.836	0.407$^{\blacklozenge}$
**ΔD_{fast}%**	-16.86 (31)	-21.93 (16)	352.000	0.891$^{\Delta}$
**PF-pre(×10^{-4})**	211.97±81.083	203.29±64.012	0.412	0.682$^{\blacklozenge}$
**PF-post(×10^{-4})**	247.50 (52)	229.50 (136)	186.500	0.207$^{\Delta}$
**ΔPF(×10^{-4})**	8.50 (73)	13.50 (154)	298.000	0.289$^{\Delta}$
**ΔPF%**	4.01 (132)	9.49 (89)	287.000	0.750$^{\Delta}$
**K^{trans}-pre(×10^{-3}/min)**	999.50±773.79	820.25±436.64	-0.935	0.345$^{\blacklozenge}$
**K^{trans}-post(×10^{-3}/min)**	722.50 (455)	715.00 (772)	226.500	0.750$^{\Delta}$
**ΔK^{trans}(×10^{-3}/min)**	-139.00 (255)	-103.00 (871)	202.000	0.370$^{\Delta}$
**Δ$K^{trans}$$_{\%}$**	-15.89 (23)	-9.98 (129)	206.000	0.423$^{\Delta}$
**V_e-pre(×10^{-3})**	668.94±239.65	666.90±249.40	-0.030	0.976$^{\blacklozenge}$
**V_e-post(×10^{-3})**	905.00 (327)	920.50 (364)	211.000	0.494$^{\Delta}$
**Δ V_e (×10^{-3})**	116.96±263.97	193.95±368.92	0.805	0.425$^{\blacklozenge}$
**Δ V_e%**	22.46 (46)	32.11 (69)	219.000	0.621$^{\Delta}$
**K_{ep}-pre(×10^{-3}/min)**	1261.50 (839)	1051.50 (909)	279.500	0.169$^{\Delta}$

**K_{ep}-post(×10^{-3}/min)**	745.00 (754)	872.50 (584)	212.000	0.509^{Δ}
**Δ K_{ep} (×10^{-3}/min)**	-343.00 (450)	-243.00 (929)	215.000	0.556^{Δ}
**Δ K_{ep} %**	-32.97 (30)	-21.99 (101)	203.000	0.383^{Δ}

Abbreviations: Diameters of tumor (cm); V: volume of tumor (cm^3); ADC: apparent diffusion coefficient(×$10^{-6}$$mm^2$/s), MD: mean diffusion (×$10^{-6}$$mm^2$/s), MK: mean kurtosis (×10^{-6}), D_{slow}: true diffusion coefficient(×$10^{-6}$$mm^2$/s), D_{fast}: pseudo diffusion coefficient(×10^{-4} mm^2/s), PF: perfusion fraction(×10^{-4}), K^{trans}: volume transfer constant (×10^{-3}/min),V_e: extracellular extravascular space(×10^{-3}), K_{ep}: rate constant(×10^{-3}/min). Statistical analysis was performed using the independent-samples t test were marked with "$^{\Delta}$" (data were reported as mean values ± standard error), while using Mann-Whitney U test were marked with" ♦"(data were presented as median (interquartile range)).
