# Supplementary material for: Comparison of the pre-treatment functional MRI metrics’ efficacy in predicting Locoregionally advanced nasopharyngeal carcinoma response to induction chemotherapy
Source: Cancer Imaging. 2021 Nov 10;21:59. doi: 10.1186/s40644-021-00428-0 (PMC8579637; doi:10.1186/s40644-021-00428-0)
Supplement: Supplementary file 3 — Additional file 3. . Pair wise comparison of ROC curves [file 40644_2021_428_MOESM3_ESM.pdf]

Pair wise comparison of ROC curves.

	**ADC-pre**	**MD-pre**	**MK-pre**	**Dslow-pre**
**ADC-pre**		0.541	0.012	0.064
**MD-pre**	0.541		0.016	0.098
**MK-pre**	0.012	0.016		0.636
**Dslow-pre**	0.064	0.098	0.636	

Abbreviations: ADC: apparent diffusion coefficient ($\times 10^{-6} mm^2/s$), MD: mean diffusion ($\times 10^{-6} mm^2/s$), MK: mean kurtosis ($\times 10^{-6}$), D_{slow}: true diffusion coefficient ($\times 10^{-6} mm^2/s$).
